# Variant pulmonary vein anatomy detected by cardiac MRI may predict outcome after pulmonary vein isolation in patients with atrial fibrillation

**DOI:** 10.1186/1532-429X-14-S1-P202

**Published:** 2012-02-01

**Authors:** Christian Mahnkopf, Manfred Duecker, Stefan Holzmann, Guido Ritscher, Oliver Turschner, Helge Simon, Johannes Brachmann, Anil-Martin Sinha

**Affiliations:** 1 Dept. of Cardiology, Klinikum Coburg, Coburg, Germany

## Background

Cardiac MRI (cMRI) has become an effective and feasible tool to identify the anatomy of the left atrium and the pulmonary veins (PV) prior to pulmonary vein ablation (PVI) in patients with atrial fibrillation (AF). It is still difficult to predict the potential success of a PVI.

## Methods

254 patients (176 male, 62.3±10.5 years) with AF were included into this study. All patients received a cMRI angiography (Siemens Verio 3T, Siemens Erlagen, Germany) of the PVs prior to PVI. All patients received at least one follow-up visit after a 90-day blanking period. Recurrent AF was defined as any symptomatic or asymptomatic detected episode lasting longer than 30 seconds.

## Results

**Figure 1:**
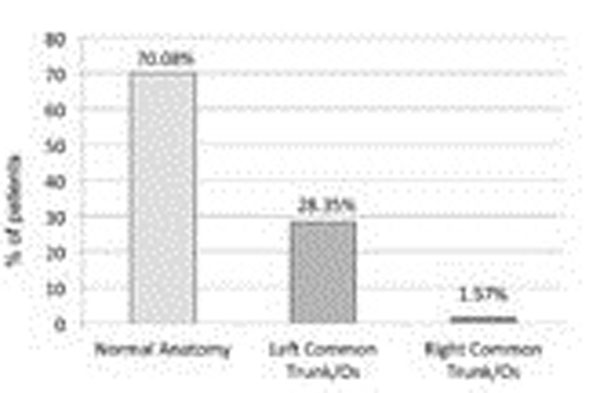
Percentage of patients with normal anatomy, left common trunk/ostium and right common trunk/ostium.

**Figure 2:**
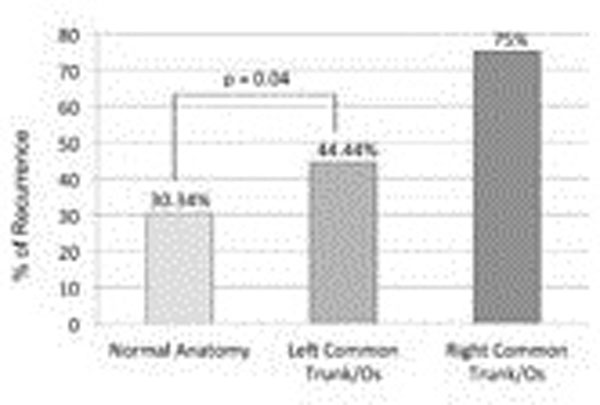
Recurrence rate in patients with normal anatomy, left common trunk/ostium and right common trunk/ostium.

Normal anatomy of the PVs (2 left, 2 right) was found in 178 patients (70.08%), whereas a common trunk/ostium of the left PVs was detected in 72 patients (28.35%), with a common trunk of the right PVs was detected in 4 patients (1.57%, Figure 1). Mean follow-up time was 136 days. Recurrent AF was detected in 54 patients (30.34%) with normal anatomy, in 32 patients (44.44%) with common trunk of the left PVs (p=0.04, Figure 2) and 3 patients (75%) with common trunk of the right PVs.

## Conclusions

From our preliminary results, anatomical variations of the PVs are common and can be detected precisely using cardiac MRI. This determination of the PV anatomy using cardiac MRI could improve the prediction of success rate after PVI as patients with a variant anatomy of the PVs appears to have a worse outcome.

## Funding

None.

